# Glucose Oxidase/Nano-ZnO/Thin Film Deposit FTO as an Innovative Clinical Transducer: A Sensitive Glucose Biosensor

**DOI:** 10.3389/fchem.2020.00503

**Published:** 2020-07-15

**Authors:** Padideh Naderi Asrami, Parviz Aberoomand Azar, Mohammad Saber Tehrani, Sayed Ahmad Mozaffari

**Affiliations:** ^1^Department of Chemistry, Science and Research Branch, Islamic Azad University, Tehran, Iran; ^2^Thin Layer and Nanotechnology Laboratory, Institute of Chemical Technology, Iranian Research Organization for Science and Technology (IROST), Tehran, Iran

**Keywords:** covalent GOx immobilization, direct current magnetron sputtering, disposable glucose biosensor, medicinal chemistry, clinical chemistry

## Abstract

In the present research, a new biocompatible electrode is proposed as a rapid and direct glucose biosensing technique that improves on the deficiencies of fast clinical devices in laboratory investigations. Nano-ZnO (nanostructured zinc oxide) was sputtered by reactive direct current magnetron sputtering system on a precovered fluorinated tin oxide (FTO) conductive layer. Spin-coated polyvinyl alcohol (PVA) at optimized instrumental deposition conditions was applied to prepare the effective medium for glucose oxidase enzyme (GOx) covalent immobilization through cyanuric chloride (GOx/nano-ZnO/PVA/FTO). The electrochemical behavior of glucose on the fabricated GOx/nano-ZnO/PVA/FTO biosensor was investigated by *I*-*V* techniques. In addition, field emission scanning electron microscopy and electrochemical impedance spectroscopy were applied to assess the morphology of the modified electrode surface. The *I*-*V* results indicated good sensitivity for glucose detection (0.041 mA per mM) within 0.2–20 mM and the limit of detection was 2.0 μM. We believe that such biodevices have good potential for tracing a number of biocompounds in biological fluids along with excellent accuracy, selectivity, and precise analysis. The fast response time of the fabricated GOx/nano-ZnO/PVA/FTO biosensor (less than 3 s) could allow most types of real-time analysis.

## Introduction

During recent decades, the determination of blood glucose level has become essential and routine because of the rapid increase in the incidence of diabetes mellitus and associated diseases. Hypoglycemia (low blood glucose level) and hyperglycemia (high blood glucose level) seriously affect the daily life of patients with diabetes (Gavin, 2007; Shabnam et al., 2017; Jedrzak et al., 2018a,b; Salek-Maghsoudi et al., 2018).

Diabetes, which is characterized by persistent hyperglycemia in clinical medicine, is one of the most important causes of death and amputation, affecting over 100 million people worldwide (Rivas et al., 2007; Zhu et al., 2016; Anderson et al., 2017). Some routine clinical laboratory checks for glucose levels, which have become an integral part of diabetes care, suffer from several limitations in accuracy, such as strip manufacturing variation, strip storage, and aging. In addition, the accuracy might be affected by environmental factors, such as temperature and altitude, and human factors, such as improper hand washing, altered hematocrit, and naturally occurring interfering substances. Last but not least, exogenous interfering substances may cause errors in the systematic measurement of blood glucose. Consequently, diabetes research centers, clinical laboratories, and medical equipment manufacturers all around the world are working toward the development and design of advanced and domestic glucose-checking devices with improved accuracy (Baghayeri, 2015; Baghayeri et al., 2017, 2020). Plenty of methods have therefore been proposed to monitor blood glucose concentration (Mozaffari et al., 2014; Rahmanian and Mozaffari, 2015; Rahmanian et al., 2015).

Among the most successful and reliable diagnostic devices are the glucose electrochemical biosensors that determine the glucose blood level based on glucose oxidase (GOx) using amperometric, impedimetric, conductometric, and potentiometric techniques. Thus, both a steady platform for enzyme immobilization and electroanalytical characterization of the fabricated sensor can guarantee the accuracy of the glucose level investigation (Miwa et al., 1994; Morikawa et al., 2002; Li et al., 2008; Wang et al., 2008; Bollella et al., 2017). There are many medical research reports on enzymatic glucose measurements based on the best-nanostructured metal oxide textures and composites (Karimi-Maleh et al., 2019c). Some nanostructured metal oxides such as zinc oxide (ZnO) have been studied in recent years owing to their strong ability to facilitate electron transfer between the electrode and the surface active site (Aini et al., 2015; Baghizadeh et al., 2015; Gallay et al., 2016; Zhao et al., 2016; Chung et al., 2017; Israr-Qadir et al., 2017; Moghaddam, 2017; Zhou et al., 2017; Karimi-Maleh et al., 2019a,c; Miraki et al., 2019; Shamsadin-Azad et al., 2019; Karimi-Maleh and Arotiba, 2020). The application of nanomaterials, especially nanoparticles, in various industries and technologies, and especially in the design of electrochemical sensors, has been well-studied (Rayati and Malekmohammadi, 2016; Dehhaghi et al., 2019; Hassandoost et al., 2019; Hosseini et al., 2019; Karimi-Maleh et al., 2019b, 2020a,b,c; Malekmohammadi et al., 2019). The direct current (DC) magnetron sputtering system is the main strategy for the production of nanostructured ZnO because of its strong capacity created by various materials for fabrication of thin films of metals, alloys, and compounds with thicknesses up to 5 p.m.

The advantages of deposition by DC magnetron sputtering system have been documented in many research papers; for example, its high purity; high adhesion film production; proper coverage by high deposition rates; deposition of any metals, alloys, or compounds; suitable porosity; and preparation of considerable volume-to-surface ratios have been intensely discussed in the literature (Panzner et al., 1985).

In this study, we develop a powerful glucose biosensor using a covalent immobilization strategy of GOx on a ZnO–polyvinyl alcohol (PVA) composite film (through cyanuric chloride) that enhances enzyme stability and the biological activity of the biomolecule. The PVA was initially deposited on fluorinated tin oxide (FTO) by a spin-coating technique (PVA/FTO), followed by DC magnetron sputtering to fabricate a nano-ZnO/PVA/FTO electrode.

The spin coating technique employed for PVA deposition is a procedure used for homogeneous thin film preparation on smooth substrates. Compared with other preparation methods, the spin coating method for the deposition of films has some significant advantages, e.g., low cost of materials and simplicity of technological runs and equipment. In this work, PVA with hydroxyl functional groups exposed on the sensor surface and many other benefits such as water-soluble organic additive activity and an electrostatic repulsive layer for anionic interference was applied to achieve ZnO/PVA nanostructures that can be used as excellent reactive sites for GOx immobilization via covalent linking.

To the best of our knowledge, for the first time, glucose determination was carried out by a unique voltammetric method using a GOx/nano-ZnO/PVA/FTO biosensor, which demonstrated a powerful ability for real sample analysis with a low detection limit (2.0 μM).

## Experimental

### Chemicals

The FTO (resistivity = 15 Ω cm^−2^) was purchased from Dyesol Company and used for fabrication of the working electrode. The FTO was washed with deionized (DI; resistivity = 18 MΩ) water, a detergent solution, and ethanol in an ultrasonic bath. PVA (MW 88 000–97 000) was purchased from Alfa Aesar Company. d(+)-Glucose was purchased from Merck Company. GOx (G6125-10KU from *Aspergillus niger*, type II, 15 000–25 000 units g^−1^) was purchased from Sigma-Aldrich. K_2_HPO_4_ and KH_2_PO_4_ were purchased from Sigma-Aldrich Co. and used for the preparation of phosphate-buffered saline (PBS; 7.4). A 100-unit solution of GOx in PBS (0.1 M, pH 7.4) was prepared as the stock solution of enzyme and kept at 4°C. A low-concentration glucose solution was freshly prepared for each measurement.

### Apparatus

In this work, a Mettler Toledo TGA 850 instrument was used for thermogravimetric analysis (TGA). Surface analysis of the fabricated sensor after the coating process was carried out by means of field emission scanning electron microscopy (FE-SEM) (TESCAN, Czech Republic). A spectrophotometer (Lambda 25 UV-Vis; PerkinElmer, USA) was used for evaluation of the absorbance factor. A DC magnetron sputtering system (The Nanostructured Coatings Co., Tehran, Iran) was used to fabricate nano-ZnO thin films with a 99.999% pure ZnO target under different experimental conditions. A 302N Autolab PGSTAT (Eco Chemie, The Netherlands) machine was used for all electrochemical assessments. GOx/nano-ZnO/PVA/FTO (A = 1 cm^2^), saturated calomel electrode (SCE), and Pt electrode were used as the working, reference, and counter electrodes, respectively. A 10 mV peak-to-peak AC amplitude was selected for electrochemical impedance spectroscopy (EIS) assessment with scanning frequencies ranging from 100 kHz to 10 MHz using the Zview/Zplot software (Macdonald et al., 1982).

## Results and Discussion

### Fabrication of the GOx/nano-ZnO/PVA/FTO Electrode

**Scheme 1:**
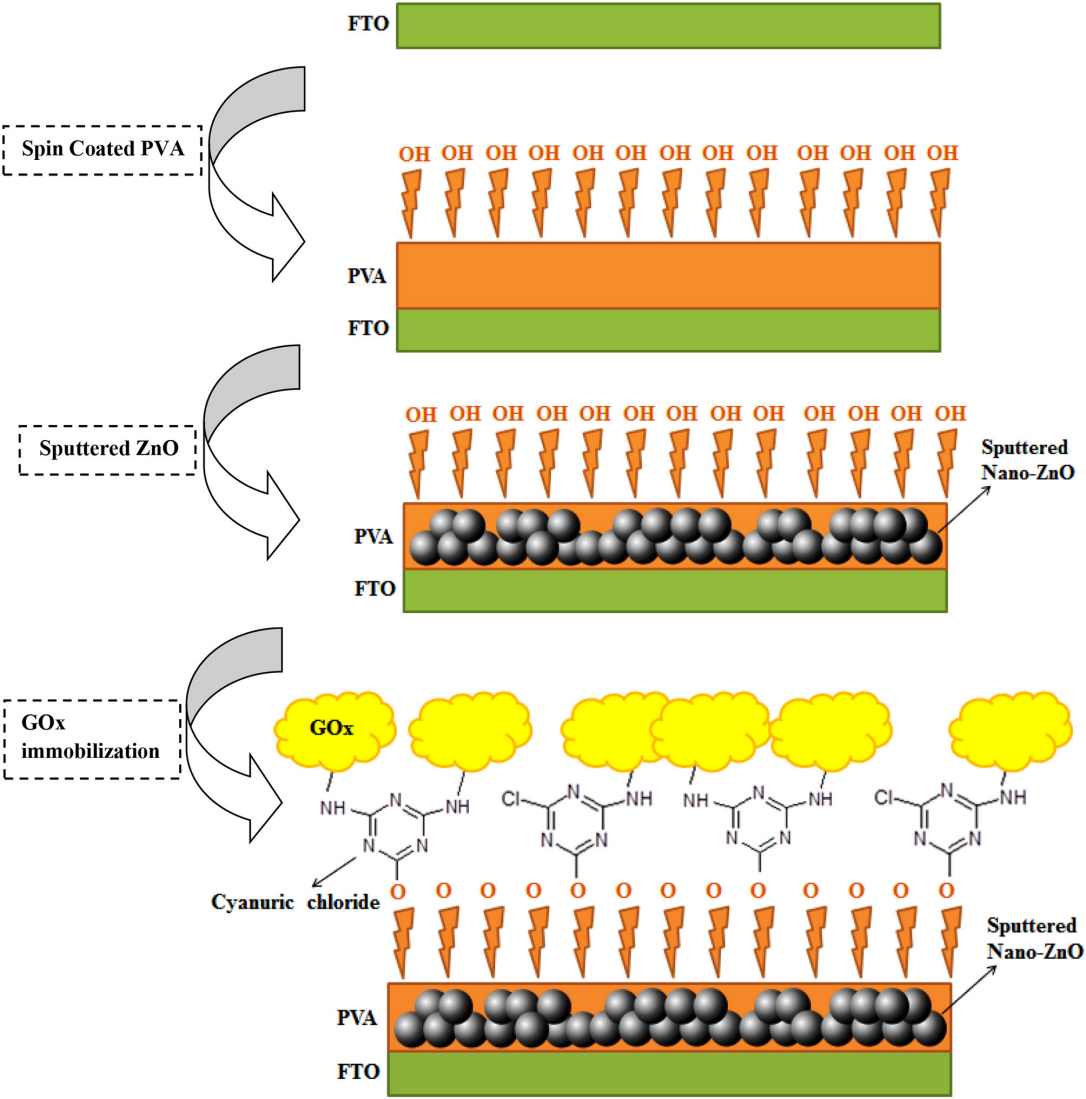
Step-by-step design of the glucose oxidase (GOx)/nano-ZnO/polyvinyl alcohol (PVA)/fluorinated tin oxide (FTO) biosensor.

The surface modification and engineering of the GOx/nano-ZnO/PVA/FTO electrode is shown in Scheme 1. The step-by-step surface engineering process will be discussed in more detail below. The current–voltage *(I-V)* results confirm successful modification of FTO by GOx and the nano-ZnO/PVA composite.

### Fabrication of PVA/FTO by Spin Coating

Because of its high electron density, affording further electrical conductivity to the ZnO film transducer and water solubility, PVA was selected as the substrate for fabrication of the biosensor. First, we optimized the spin coating parameters, such as spinning speed, spinning duration, solution volume, and PVA content, for the electrical resistivity. The resistivity of a PVA/FTO surface fabricated by the spin coating technique was measured by using a four-point collinear probe in the air. Four sets of deposition parameters—spinning speed (200–2,400 rpm), spinning duration (10–60 s), solution volume (0.1–0.9 mL), and precursor PVA content (1–10 g L^−1^)—were investigated in order to obtain a uniform and suitable thin film. In each set of samples, only one of the parameters mentioned above was varied while the other parameters were kept constant. Spinning speed has been shown to have substantial effects on many physical properties, such as film thickness and mechanical properties. The spin coating conditions were chosen to be optimal for PVA thin film formation with the best electrical resistivity in this work.

**Figure 1:**
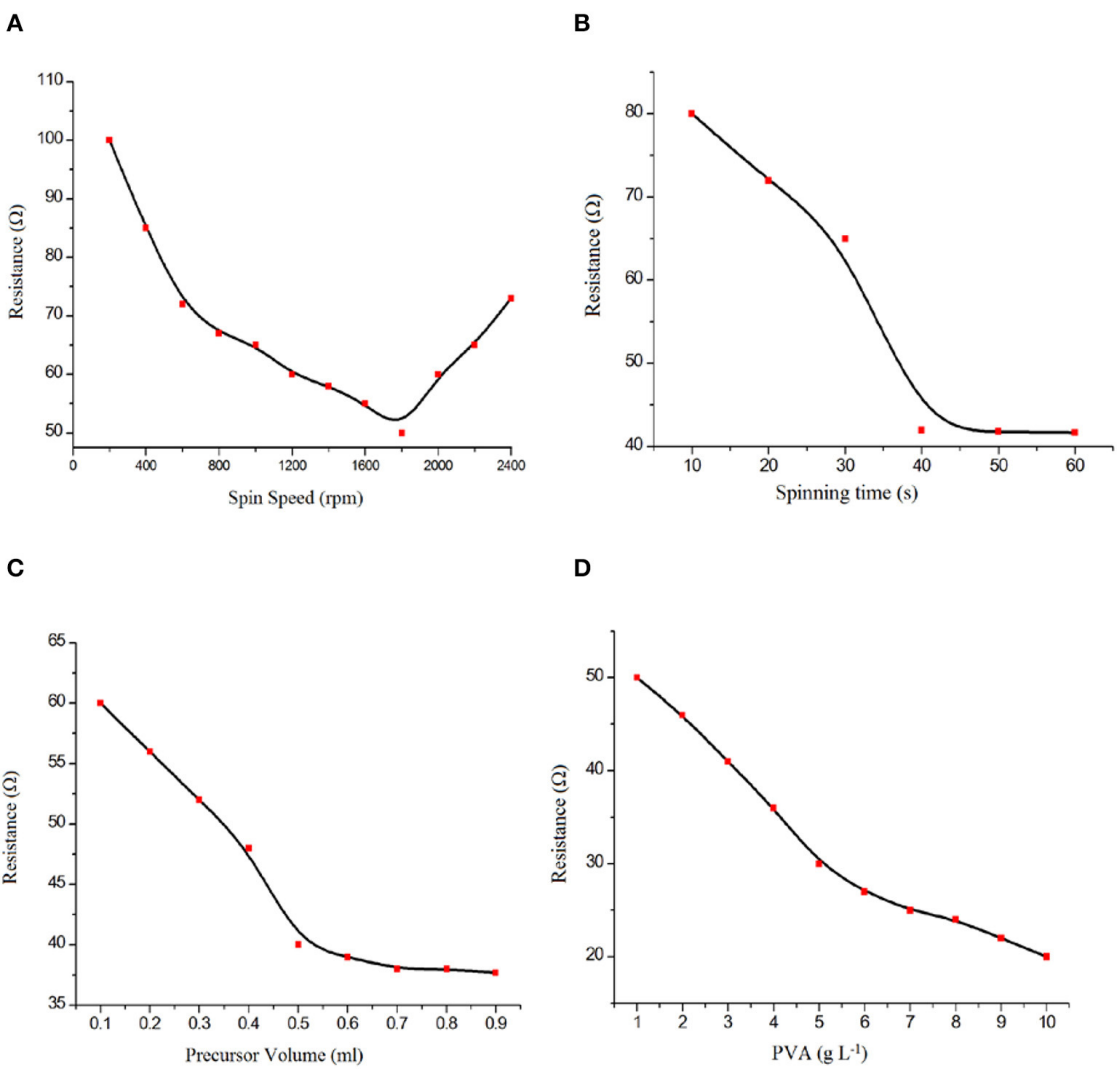
Effect of **(A)** spin speed, **(B)** spinning time, **(C)** precursor volume, and **(D)** polyvinyl alcohol (PVA) content on PVA/fluorinated tin oxide (FTO) characteristics.

Figure 1A shows the relation between the PVA thin film electrical resistivity and the spinning speed (200–2,400 rpm). Increasing the spinning speed to 1,750 rpm results in a decrease in the electrical resistivity of the thin film. Decreasing the film thickness by decreasing the spinning speed to 1,750 rpm results in a uniform PVA structure while increasing the spinning speed to 2,500 rpm results in a non-uniform PVA thin film because of the high centrifugal force. The result shows a non-linear relationship between the spinning speed and electrical resistivity of the thin film. A spinning speed of 1,750 rpm was selected as the optimum spinning speed.

Figure 1B shows that the electrical resistivity is improved by an increase in spinning duration from 10 to 40 s, while there was no noticeable change after 40 s. During the first few seconds of the spin coating process, a small portion of the substrate is covered by PVA as an electrically conductive layer. After 40 s, the spinning duration did not have any significant effect on the electrode resistance. Therefore, the optimum spinning time was selected as 40 s.

Figure 1C shows that the resistivity is decreased with an increase in the volume of the deposition solution from 0.1 to 0.7 mL, while there was no noticeable change after 0.9 mL. Thus, 0.7 mL was selected as the optimum volume of the solution for economic reasons.

The precursor PVA content is an effective parameter in the spin coating process. The PVA content not only results in a thin film with lower electrical resistivity but also prepares suitable –OH groups in the substrate for GOx covalent immobilization. By increasing the amount of PVA content in the precursor, a lower electrical resistivity of the thin film has been observed (Figure 1D). Furthermore, the precursor PVA content has a significant effect on GOx immobilization, so a precursor concentration of 10 g L^−1^ was selected.

In summary, 1,750 rpm (spinning speed), 40 s (spinning duration), 0.7 mL (the volume of solution), and 10 g L^−1^ (PVA content) were selected as the optimum conditions for the spin coating procedure.

### Fabrication of nano-ZnO/PVA/FTO

The nano-ZnO was deposited on the surface of a PVA/FTO electrode using reactive DC magnetron sputtering under an Ar: O_2_ gas atmosphere while the resistivity was measured by a four-point collinear probe in the air. The Zn cathode plate was bombarded by Ar^+^ ions generated in the glow discharge plasma placed in front of the Zn target in the evacuated sputtering chamber. A specific amount of O_2_ gas is used for metal-oxide fabrication in a reactive sputtering process.

The deposited ZnO thin film was prepared by chemical reaction of Zn atoms from the target surface and O_2_ reactive gas during reactive sputtering. The metal–metal oxide composition of the nano-ZnO film can be determined by control of the pressure of Ar and O_2_ gases (Kelly and Arnell, 2000; Baghriche et al., 2012; Bijad et al., 2013; Yao and Lu, 2013; Mozaffari et al., 2014).

The optimum conditions in the synthesis procedure were obtained by varying the sputtering experiment conditions, such as the sputtering power (100–400 W); deposition time (1–25 min); distance between the target and substrate (4–10 cm); mixed gas pressure (total gas pressure from 1 × 10^−3^ to 1 × 10^−2^ Torr); and Ar: O_2_ gas flow ratio (1:1–8:1 sccm/min) in order to obtain a very uniform ZnO thin film.

**Figure 2:**
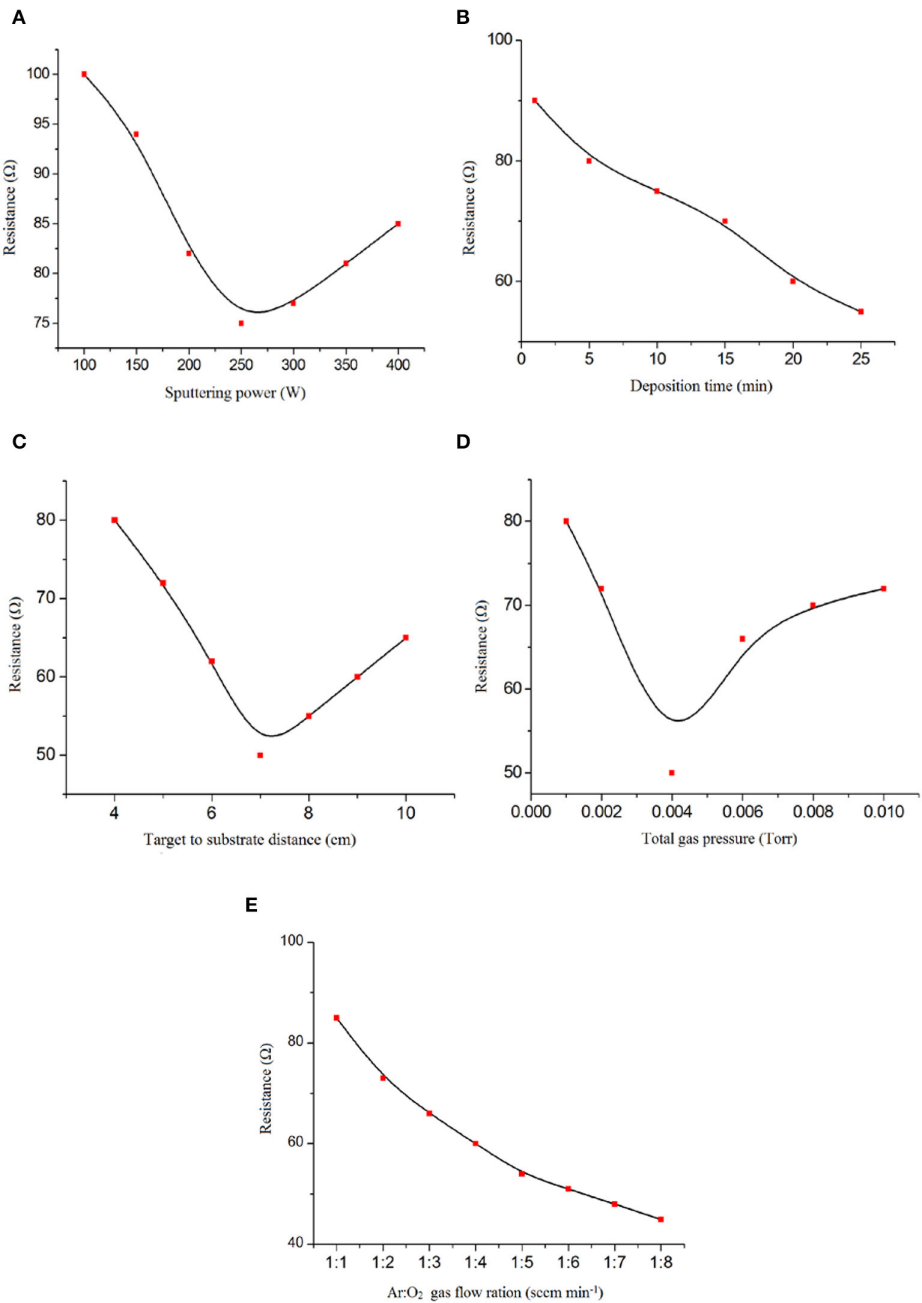
Effect of **(A)** DC magnetron sputtering power, **(B)** deposition time, **(C)** target-to-substrate distance, **(D)** total gas pressure, and **(E)** Ar: O_2_ gas ratio on nano-ZnO/polyvinyl alcohol (PVA)/fluorinated tin oxide (FTO) characteristics.

According to the data reported in Figure 2A, the DC sputtering power showed strong effects on the fabrication of the ZnO thin film. Therefore, optimization of the sputtering conditions to form nano-ZnO thin films with the lowest electrical resistivity is necessary.

As can be observed, increasing or reducing the DC sputtering power affects the amount of nano-ZnO sputtered atoms from the target to the substrate. Hence, a lower DC sputtering power leads to a lower amount of Zn sputtered atoms and increases the chance of ZnO formation, which results in higher electrical resistivity of the thin film. However, a higher DC sputtering power of up to 250 watts produced higher amounts of non-reacted Zn sputtered atoms. Additionally, oxygen deficiency in the deposited film increases and leads to lower electrical resistivity. The probable reality behind these comments could be that the PVA thin film is degraded by high-energy particle bombardment due to the excessive DC sputtering power supply, which resulted in a non-uniform thin film.

The deposition time was selected as the effective factor for obtaining a uniform nano-ZnO thin film. Lower electrical resistivity of the thin film is observed by increasing the time of deposition from 1 to 10 min (Figure 2B). With the increase in deposition time and improvement in the thickness of the thin film, we observed low thin film resistivities that are useful for this type of sensor.

The effect of distance between the target and substrate in the range 4–7 cm on the electrical resistivity of the nano-ZnO thin film is shown in Figure 2C. Because of the lower amount of nano-ZnO particles deposited, we detected an improvement in electrical resistivity of the film by increasing the distance between the target and the substrate from 4 to 7 cm. Also, thin film defection by highly energized Ar^+^ occurred. As shown in Figure 2C, a distance of 7 cm between the target and the substrate was selected as the best condition in this study.

The porosity and physical homogeneity of the ZnO thin film is directly affected by the total gas pressure during plasma formation (Figure 2D). This factor can be affected by the deposition rate, which therefore also helps to create a more homogeneous thin film. In this case, a total gas pressure in the range of 1 × 10^−3^ to 1 × 10^−2^ Torr was tested and, according to the results obtained, 1 × 10^−3^ Torr was selected as the optimum condition for total gas pressure.

The Ar: O_2_ gas flow ratio was selected as another factor affecting the preparation of a ZnO thin film with lower electrical resistivity in a reactive DC magnetron sputtering system. By decreasing the oxygen ratio in the gas flow, we detected a lower electrical resistivity of the thin film that is useful for the fabrication of GOx/nano-ZnO/PVA/FTO (Figure 2E). This term clarified the important role of the oxygen gas flow ratio in Zn: ZnO phase formation in thin film preparation.

According to the obtained results, 250 W (sputtering power), 10 min (deposition time), 7 cm (distance between target and substrate), 4 × 10^−3^ Torr (total pressure), and 8: 1 (Ar: O_2_ gas flow ratio) were selected as the optimum conditions for the preparation of a uniform ZnO thin film.

### Fabrication of GOx/nano-ZnO/PVA/FTO Biosensor

The bonding status of the biomolecule to the transducer matrix is one of the significant factors in biosensor fabrication. One serious problem is the leaching of the biomolecule out of the surface during the preparation process.

Therefore, a new design based on the reaction between cyanuric chloride as the anchor molecule and PVA as the source of free –OH groups was used. This design helps GOx to be covalently attached to the anchor by its –NH_2_ groups (Scheme 1). The nano-ZnO/PVA/FTO electrode was dipped into cyanuric chloride ethanolic solution (100 g L^−1^) for 2 h with stirring. After this step, the amplified sensor was put into 0.1 M PBS (pH 7.4) containing 100 units of GOx for 4.0 h with stirring. Step-by-step fabricated biosensor monitoring was achieved by EIS and cyclic voltammetry (CV) techniques. The *I*-*V* technique was also applied to the fabricated glucose biosensor (Ansari et al., 2008; Mahadeva and Kim, 2011; Rahmanian and Mozaffari, 2015).

### Morphological Characterization of GOx/nano-ZnO/PVA/FTO

**Figure 3:**
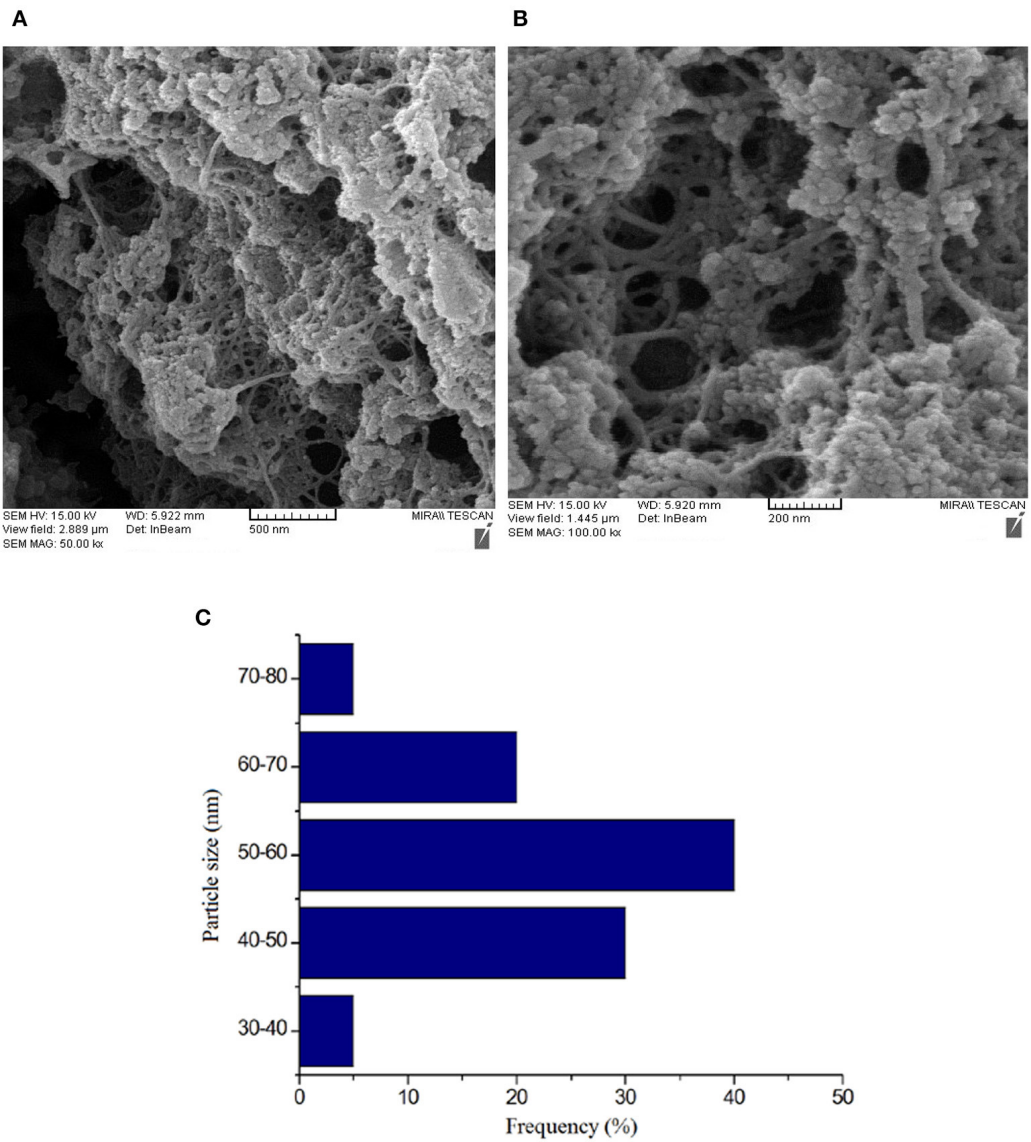
Field emission scanning electron microscopy images of **(A,B)** the nano-ZnO/polyvinyl alcohol (PVA)/fluorinated tin oxide (FTO) at different magnifications and **(C)** its size distribution histogram. The particle size abundance was 50–60 nm.

The surface morphology of ZnO/PVA/FTO and also the size distribution histogram of ZnO nanoparticles was investigated by FE-SEM. The results are presented in Figures 3A–C. The results confirm a good and uniform distribution of ZnO/PVA as a film on the surface of FTO that can play a significant role in covalent enzyme immobilization.

**Figure 4:**
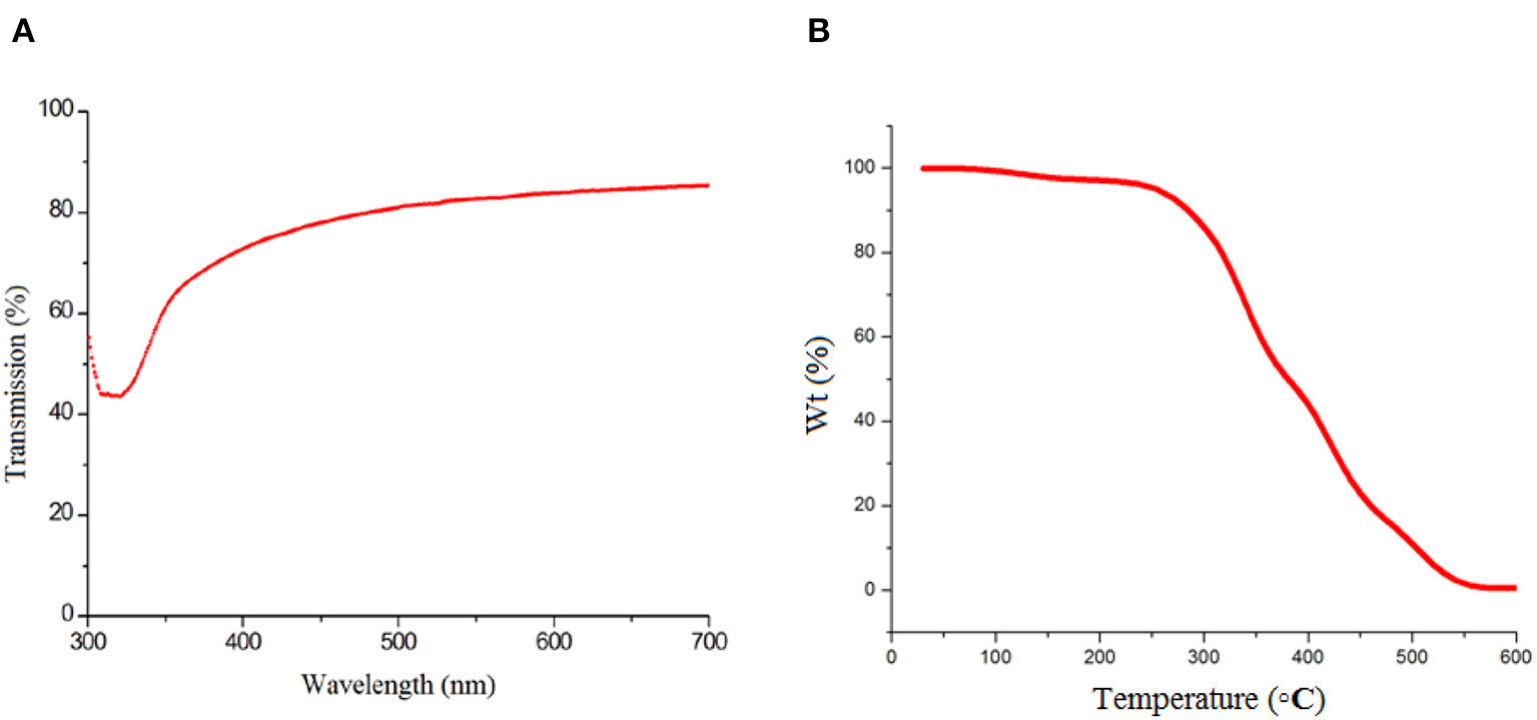
**(A)** Typical optical transmission spectra recorded for nano-ZnO/polyvinyl alcohol (PVA)/fluorinated tin oxide (FTO) and **(B)** thermogravimetric analysis of nano-ZnO/PVA/FTO.

The optical transmission (ultraviolet) spectrum of the nano-ZnO/PVA composite film was recorded and the result is presented in Figure 4A. As can be seen in Figure 4A, there is a sharp absorption band at a wavelength above 317 nm that confirms the optical quality and low concentration of defects of the nano-ZnO/PVA thin film. Additionally, the TGA technique was applied for mass loss assessment of the thin film as a function of temperature at 5°C min^−1^ and under an N_2_ atmosphere for thermal stability determination of the nano-ZnO/PVA. The ZnO/PVA mass loss curve shows the low thermal stability of the polymer, as a property common to polymers with low molecular weight. The ZnO/PVA curve exhibits two degradation stages. The first one at 327°C is related to the elimination of the amorphous parts of the PVA polymer while the second one, at 380°C, corresponds to the degradation of the crystalline parts with higher thermal stability (Figure 4B). Ultimately, the white ZnO powder remained after TGA analysis.

### Electrochemical Characterization of GOx/nano-ZnO/PVA/FTO

**Figure 5:**
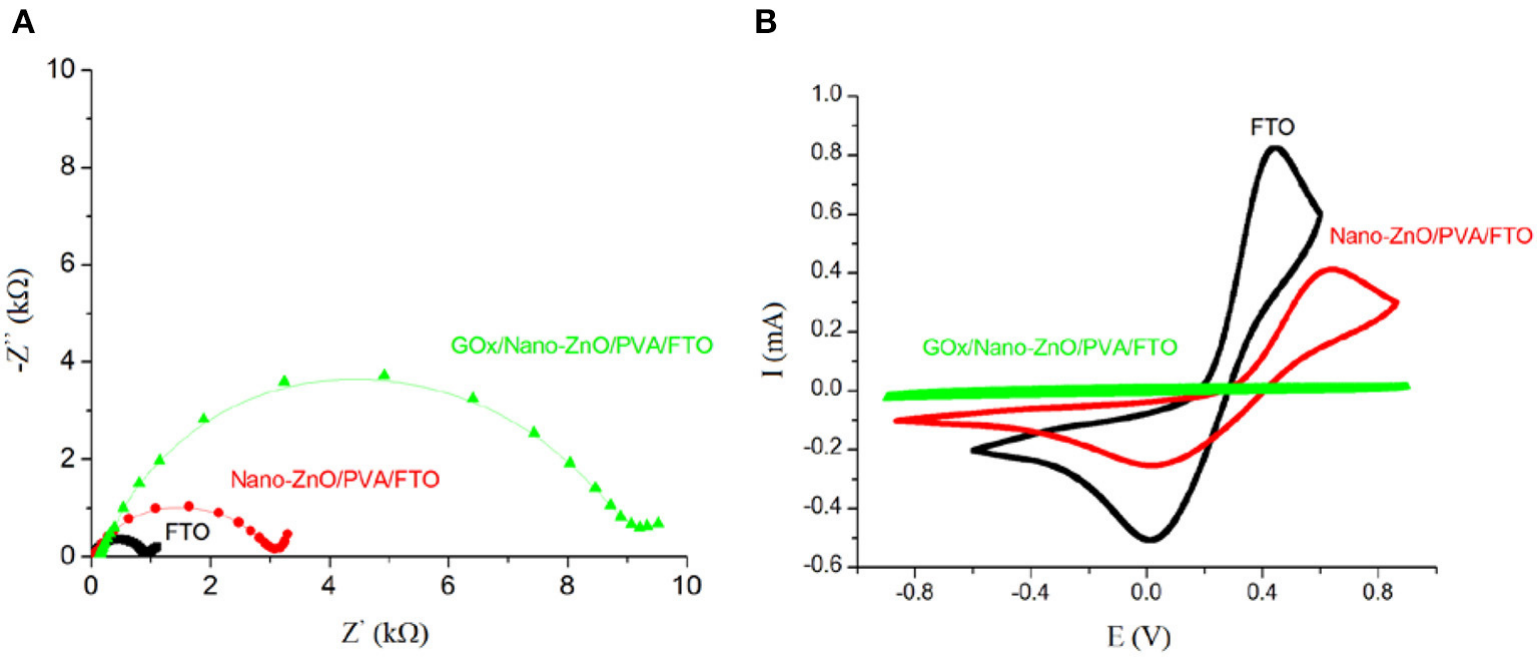
**(A)** Nyquist plots of a solution containing 5 mM [Fe(CN)_6_]^3−/4−^ at the surface of fluorinated tin oxide (FTO), nano-ZnO/polyvinyl alcohol (PVA)/FTO, and glucose oxidase (GOx)/nano-ZnO/PVA/FTO. **(B)** Cyclic voltammograms (scan rate of 0.1 V s^−1^) of a solution containing 5 mM [Fe(CN)_6_]^3−/4−^ at the surface of FTO, nano-ZnO/PVA/FTO, and GOx/nano-ZnO/PVA/FTO. (Z′ is the real Warburg impedance and Z″ is the imaginary Warburg impedance).

We used the EIS method in the presence of a redox probe ([Fe(CN)_6_]^3−/4−^) as a powerful technique to study the interfacial changes at the surface of the FTO during the modification process (Jamali et al., 2014; Mozaffari et al., 2015a,b; Tahernejad-Javazmi et al., 2018, 2019; Khodadadi et al., 2019). Using the semicircle diameter of a Nyquist plot of the modification process in a solution containing the redox probe [Fe(CN)_6_]^3−/4−^, we can study the change in the charge transfer resistance (*R*_ct_) and examine the effect of conductive mediators at the surface of the electrodes (Mozaffari et al., 2009, 2010; Ensafi and Karimi-Maleh, 2010; Karimi-Maleh et al., 2016; Shojaei et al., 2016; Asrami et al., 2017; Shamsadin-Azad et al., 2019). Figure 5A shows Nyquist plots of a solution containing 5.0 mM [Fe(CN)_6_]^3−/4−^ with 1.0 M KNO_3_ at the surface of different modified electrodes. We detected an *R*_ct_ value of 0.9 kΩ for an FTO bare electrode, which confirms that the highest conductivity of this electrode is due to its inherent electrode properties. FTO exhibits a low electrical resistivity owing to the high carrier concentration (*N*_d_) caused by the oxygen vacancies and the fluorine doping. The maximum *R*_ct_ value (9.2 kΩ) is obtained on GOx/nano-ZnO/PVA/FTO (final modified structure). The progressively increasing interfacial resistance during the surface modification is attributed to the lower electrical resistivity of the newly generated layer on FTO because of the GOx insulating characterization. Moreover, the CV study of the layer-by-layer assembly of GOx/nano-ZnO/PVA/FTO in glucose-free PBS containing 5.0 mM [Fe(CN)_6_]^3−/4−^ is shown in Figure 5B. The magnitude of the anodic peak current for the GOx/nano-ZnO/PVA/FTO electrode (0.03 mA) being lower than that for the bare FTO electrode (0.82 mA) reveals the insulating characteristics of GOx. The outcome data from CV and EIS in the modification process confirm each other.

### Electrochemical Characterization of the GOx/nano-ZnO/PVA/FTO Biosensor in the Presence of Glucose

**Figure 6:**
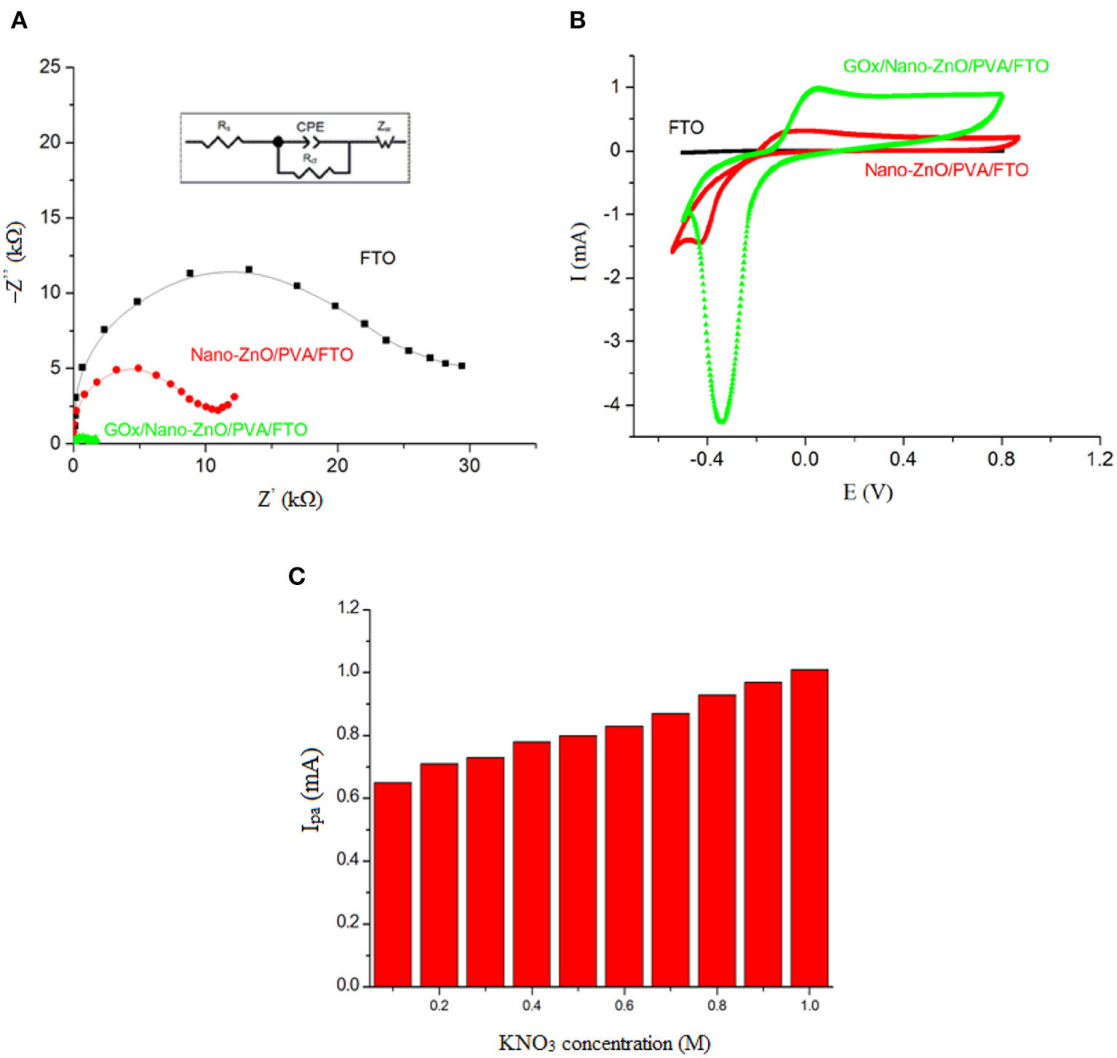
**(A)** The Nyquist plots (−Z″ vs. Z′), and **(B)** Cyclic voltammograms at the scan rate of 0.1 V s−1 obtained in PBS (pH 7.4) containing 5 mM glucose in the absence of [Fe(CN)6]3−/4− probe, and **(C)** The influence of KNO3 salt concentration, as a supporting electrolyte, on the cyclic voltammogram electrochemical response of 5 mM of glucose on GOx/Nano-ZnO/PVA/FTO biosensor. The inset in **(A)** shows equivalent circuit. (Zw: Warburg impedance, Ipa: anodic peak current, Rs: solution resistance, Rct: charge transfer resistance).

The electrochemical behavior of the layer-by-layer assembly of the biosensor was verified in the presence of 5.0 mM glucose and 1.0 M KNO_3_. Figure 6A shows the recorded levels relative to the EIS investigation in the presence of glucose to determine the *R*_ct_ value of the interfacial properties of the GOx/nano-ZnO/PVA/FTO. For reliable impedimetric studies with unchanging conditions, we selected an open circuit potential (OCP) −0.1 V system. The other reason for choosing a −0.1 V DC potential is related to *R*_ct_, which is the kinetic component of the resistance determined by EIS. Therefore, to obtain a kinetic-controlled interfacial process, the DC potential was selected to match the kinetic region of the voltammogram in Figure 6B.

The *R*_ct_ value in the presence of glucose for the nano-ZnO/PVA/FTO (9.71 kΩ) is lower than that for the FTO electrode (28.80 kΩ), which is due to a small direct glucose reaction with FTO. A further reduction in the *R*_ct_ value (1.61 kΩ) was observed after immobilization of GOx onto the nano-ZnO/PVA/FTO matrix owing to rapid glucose oxidation by the GOx, which can represent the faster, predictable, and reproducible response of GOx/nano-ZnO/PVA/FTO compared with the slower, unpredictable, and non-reproducible response of ZnO. The EIS analysis was carried out by means of a modified Randles equivalent circuit, where the constant phase element (CPE) is in series with the Warburg impedance (*Z*_w_), in parallel with the *R*_ct_, and in series with the solution resistance (*R*_s_) (Figure 6A, inset). Cyclic voltammetric studies of the layer-by-layer assembly of GOx/nano-ZnO/PVA/FTO in PBS containing 5.0 mM glucose are shown in Figure 6B. The CV results support the EIS findings, where the cathodic peak current refers to the O_2_ + 2e^−^ → 2O^−^ reaction accompanied by the H_2_O_2_ → O_2_ + 2H^+^ + 2e^−^ reaction for the anodic peak current. The mechanism is explained in detail below.

According to the Henry and Peter microscopic model, sensors researchers described the changing electrical conductance of a semiconducting film sensor in a solution containing toxic gases (Guilbault and Lubrano, 1973; Windischmann and Mark, 1979; Ansari et al., 2008). According to the microscopic model, in the first step, the atmospheric oxygen molecules could be adsorbed at surface sites by physical bonding. According to this reaction, atmospheric oxygen molecules convert to the ion form and are then adsorbed at the surface of the system as ${\text{O}}_{\text{ads}}^{-}$. The resulting equation is:

(1) $$\begin{array}{l} {\text{O}}_{2}+2{\text{e}}^{-}\to 2{\text{O}}^{-} \end{array}$$

Equation (1) leads to a decrease in the conductance of the transducer, as indicated by an increase in the potential barrier at the grain boundaries, upon exposing GOx immobilized to glucose, the reaction between GOx and glucose can be described according to the below equations:

(2) $$\begin{array}{l} \text{Glucose}+{\text{O}}_{2}\to \text{gluconolactone}+{\text{H}}_{2}{\text{O}}_{2} \end{array}$$

(3) $$\begin{array}{l} {\text{H}}_{2}{\text{O}}_{2}\to {\text{O}}_{2}+2{\text{H}}^{+}+2{\text{e}}^{-} \end{array}$$

This results in the production of d-gluconate with an H^+^ ion, which reacts with ${\text{O}}_{\text{ads}}^{-}$ and thereby releases the trapped electron to the conduction band of ZnO, as indicated by the decrease in the potential barrier at the grain boundary. Further, when reducing molecules (R: H_2_O_2_) react with preadsorbed negatively charged oxygen adsorbates, the trapped electrons are returned to the conduction band of the material. The energy released during the decomposition of adsorbed molecules would be sufficient for the electrons to jump up into the conduction band in such a way that it increases the conductivity of the biosensor based on the following reaction:

(4) $$\begin{array}{l} \text{R}+{\text{O}}^{-}\to \text{RO}+{\text{e}}^{-} \end{array}$$

However, some valuable proposed photoelectric mechanisms is interesting for researchers and can prepare a vast view in the semiconductors researches (Liang et al., 2017).

### Effect of Salt Concentration and pH of Solution

Because of the maximum activity of GOx enzyme at pH 7.4, this pH value was selected as the optimum value for glucose oxidation in the present study. It must be noted that the survival of the enzyme's natural structure is necessary to improve the detection limit and sensitivity for glucose detection. PVA possesses a negative charge surface, which repels the negatively charge GOx [with low isoelectric point (IEP) 4.5] at physiological pH (pH = 7.4), so covalent GOx immobilization to the substrate was required.

Using supporting electrolytes in an electrochemical process is necessary to eliminate the transport of electroactive species by ion migration in an electric field and to increase the conductivity of the solution. The selection of a suitable supporting electrolyte with special properties such as electrochemical stability; solubility; interaction with the reaction intermediate; and some preparation difficulties is very important in a voltammetric investigation. Different types of water-based buffers were used as inorganic supporting electrolytes. In this case, the test solutions, such as KClO_4_, NaNO_3_, KCl, KNO_3_, and KI, were also used to examine the effects of the supporting electrolytes at different concentrations (concentration changed between 0.0 and 1.0 M) on the voltammogram signals of 5.0 mM glucose. The outcomes showed that the sensor sensitivity and the oxidation signal of glucose were enhanced by increasing the concentration of the salt to 1.0 M KNO_3_ as a supporting electrolyte (Figure 6C) and this supporting electrolyte was selected as the best condition for the following steps.

### The Working Curve for Ohmic Behavior Measurements

The catalytic activity of the GOx/nano-ZnO/PVA/FTO was determined under the following conditions: a DC current cell and a gold wire (5 cm length, Φ 0.3 mm) as an electrode and the GOx/nano-ZnO/PVA/FTO as a working electrode were used in a potential range of 0.0–1.0 V. At higher voltages, it is possible to oxidize water as a solvent. In water electrolysis, the oxygen will bubble at the anode.

**Figure 7:**
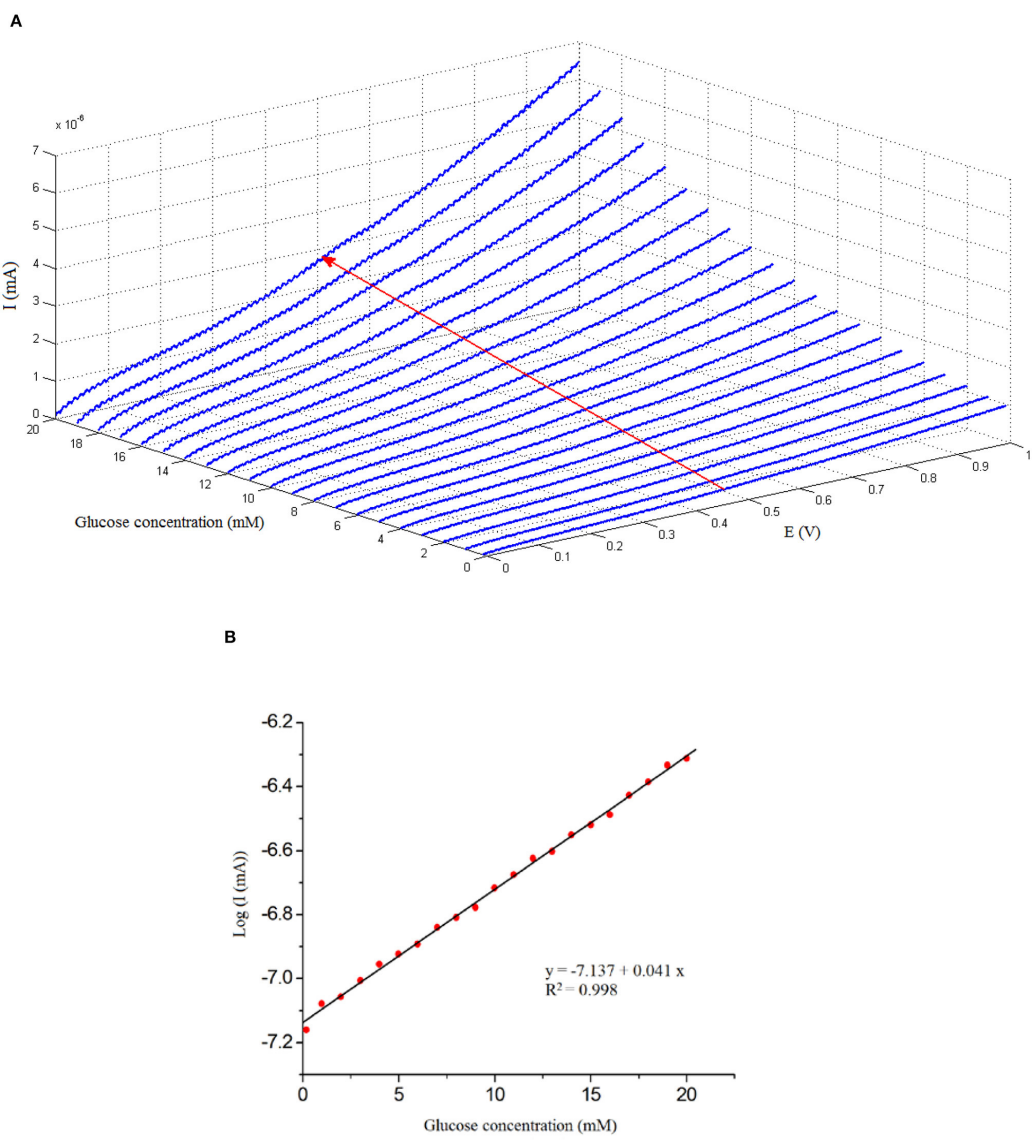
**(A)** Current–voltage (*I*-*V*) plots obtained on the GOx/nano-ZnO/polyvinyl alcohol (PVA)/fluorinated tin oxide (FTO) biosensor in the presence of different concentrations of glucose: 0.2–20 mM with a rate of 0.1 V/s. **(B)** Variation of *I*-*V* plots current as a function of glucose concentration, at *E* = 0.5 V, which represents the calibration curve (*y* = −7.137 + 0.041*x, R*^2^ = 0.998).

As can be seen in Figure 7A, different concentrations of glucose solutions, such as 0.2–20 mM, were prepared in 50 mL of PBS electrolyte at 25°C. The effect of increasing the glucose concentration is specified in the *I*-*V* curve (Figure 7A).

The increase in the slope of the *I*-*V* curve (Δ*I*/Δ*V*) indicates successful enzyme immobilization. The proposed equation for sensitivity determination is: sensitivity = (Δ*I*/Δ*V*)_*x*_ – (Δ*I*/Δ*V*)_0_/(Δ*I*/Δ*V*)_0_, where (Δ*I*/Δ*V*)_0_ is the *I*-*V* curve's slope at a glucose concentration of “0.0” mM and (Δ*I*/Δ*V*)_*x*_ is the slope of the *I*-*V* curve at a glucose concentration of “*x*” mM, where *x* = 0.2–20 mM of glucose.

The volume of 50 mL of electrolyte was chosen in all of the electrochemical measurements. Since the amount of glucose with respect to PBS changes with increasing molar concentration, this effect would be reflected in the Δ*I*/Δ*V* of the *I*-*V* curves, as presented in Figure 7A. Enhancement in the sample current is noticeable, i.e., the film conductivity increases. This enhancement is attributed to the catalytic reaction of GOx and glucose:

$$\begin{array}{l} \text{Glucose}+{\text{O}}_{2}\to \text{gluconolactone}+{\text{H}}_{2}{\text{O}}_{2} \end{array}$$

The sensitivity (Δ*I*/Δ*V*) increases promptly with the increase in glucose concentration because of the increase in the hydrolysis of glucose. The resistance of GOx/nano-ZnO/PVA/FTO decreases in the presence of glucose. Obviously, H_2_O_2_ increases with the increase in glucose, and more and more trapped electrons are returned to the conduction band of the biosensor, which explains the increase in sensitivity of the sensor (partially) linearly (ohmic behavior) with glucose concentration. The high activity of GOx/nano-ZnO/PVA/FTO is due to the high surface area of the thin film.

The *I*-*V* investigation confirms a reversible system and supports the catalytic activity of GOx at the surface of GOx/nano-ZnO/PVA/FTO. The linear dynamic range investigation results for the determination of glucose using a GOx/nano-ZnO/PVA/FTO biosensor are shown in Figure 7B. *I*-*V* responses in Figure 7B shows a working curve in a linear scale [log *i* (mA) = −7.137 (±0.005) + 0.041 (±0.001) × [glucose] (mM); *R*^2^ = 0.998] in a concentration range of 0.2–20 mM of glucose in *E* = 0.5 V. The GOx/nano-ZnO/PVA/FTO biosensor exhibits a detection limit of 2.0 μM for glucose and a sensitivity of 0.041 mA per mM with a linear range of 0.2–20 mM.

In addition, the impedimetric signal of nano-ZnO/PVA/FTO vs. the reducing (R) molecule (H_2_O_2_, in the absence of glucose) concentration was recorded as new evidence to validate our proposed biosensing mechanism. According to the data (were not shown), there was a significant decrease in the *R*_ct_ value after the addition of H_2_O_2_ from 0.001 to 0.021 M. This phenomenon is due to the reaction between the reducing species and the preadsorbed negatively charged oxygen adsorbates (Equation 3). This reaction results in trapped electrons being returned to the conduction band of ZnO, and as a result a decrease in *R*_ct_ is observed. The obtained results in the impedimetric investigation in the presence of H_2_O_2_ species support the proposed mechanism.

### Analytical Characteristics

The ZnO thin film (IEP ~9.5; pH = 7.4) possesses a positive charge surface that repulses positive metal ions; as a result, quantitative analysis in the presence of metal ions (including Zn^2+^, Mn^2+^, Ca^2+^, Ag^+^, Cu^2+^, Al^3+^, Mg^2+^, and Pb^2+^) was possible. Furthermore, the selectivity of GOx/nano-ZnO/PVA/FTO toward the determination of glucose (5 mM) was investigated by an electrochemical technique by adding the usual concentration of interfering substances that are present in human serum such as uric acid (0.1 mM) and ascorbic acid (0.1 mM). The obtained *I*-*V* data show a maximum interference of 1.5% in the presence of all of the interfering substances, which confirms the selectivity of GOx/nano-ZnO/PVA/FTO as a glucose biosensor.

Numerous assays have been conducted on blood serum to examine the ability of the GOx/nano-ZnO/PVA/FTO biosensor to determine glucose levels in real-life samples using a calibration curve. The results obtained by the proposed method were checked with a spectrophotometric method by a local clinical laboratory (Table 1). The results presented in Table 1 show clearly the ability of the GOx/nano-ZnO/PVA/FTO biosensor to act as a novel and powerful tool for determination of glucose in real-life samples.

**Table 1:** Measurement of the level of glucose in blood serum.

**Method**	**Sample number**				
	**1**	**2**	**3**	**4**	**5**
Determination by Spectrophotometry (mM)	4.42	5.25	4.10	3.24	6.10
Determination by GOx/Nano-ZnO/PVA/FTO biosensor (mM)	4.43	5.27	4.09	3.24	6.09
R.S.D.^a^ (*n* = 5) (%)	1.50	1.10	1.2	1.55	1.00

R.S.D.

a *Relative Standard Deviation*.

The sensor was studied by recording the CV anodic peak currents in the presence of 5.0 mM glucose and 1.0 M KNO_3_ after continuous scanning for 500 cycles, which showed that the electrode retained 96.5% of the initial current response and proved the stability of the biosensor in buffer solution (The data were not shown).

Additionally, 10 modified electrodes were utilized for parallel determinations of 5.0 mM glucose and the relative standard deviation (RSD) was determined as 1.21%, indicating that the electrode had good reproducibility for the precision study. We detected an RSD value of 1.65% for determination of 5.0 mM glucose using the same 10 GOx/nano-ZnO/PVA/FTO electrodes fabricated with the same procedure. This RSD value confirmed the good repeatability for GOx/nano-ZnO/PVA/FTO as a novel glucose biosensor. The response time of GOx/nano-ZnO/PVA/FTO was checked as an important analytical factor and results showed a fast response time (less than 3 s) for GOx/nano-ZnO/PVA/FTO in the determination of glucose, confirming the rapid rate of diffusion of the analyte into the enzyme surface and rapid reaction between the analyte and the enzyme. Furthermore, the stability of the GOx/nano-ZnO/PVA/FTO biosensor was checked by comparing the oxidation signal of glucose at different time periods. Three GOx/nano-ZnO/PVA/FTO biosensors lost about 0.0, 2.0, and 3.0% of their original activity (for a fixed glucose concentration of 5.0 mM), after storage times of 1, 30, and 60 days, respectively. Table 2 compares the analytical characteristics of the GOx/nano-ZnO/PVA/FTO biosensor with other reported glucose biosensors. The biosensor in the present work clearly shows better analytical factors, such as the detection limit and response time, than previously reported sensors (Wei et al., 2010; Marie et al., 2015; Gallay et al., 2016; Mandal et al., 2016; Asrami et al., 2018; Nguyen et al., 2019; Sabu et al., 2019). This advantage is relative to covalent enzyme immobilization in our fabricated biosensor. This covalent enzyme immobilization is the main consideration in creating good *K*_m_ and *V*_m_ factors in an enzymatic biosensor that help to improve biosensor activity.

**Table 2:** Analytical characteristics GOx/Nano-ZnO/PVA/FTO biosensor compared with the other reported ZnO base glucose biosensors.

**Electrode matrix**	**D.L (nM/μM)**	**Sensitivity**	**D.R (μM/mM)**	**RT (Sec)**	**Detection method**	**Ref**.
ZnO nanorods/Au hybrid	10 nM	48.0 μA/mM	0.1–33.0 μM	–	Amperometric/Enzymatic	Wei et al., 2010
ZnO nanorods	0.089 mM	1 mA/mM cm^−2^	1.27–16.62 mM	–	Amperometric/Enzymatic	Mandal et al., 2016
Zinc oxide nanorods	0.22 μM	10.911 mA/mM cm^−2^ 17 μA/mM cm^−2^	1.5–7 mM	3	Amperometric/Enzymatic	Marie et al., 2015
Zinc oxide nanwire	–		0.03–1.52 mM	–	Amperometric/Enzymatic	Gallay et al., 2016
GOx/Nano-ZnO/PVA/FTO	2.0 μM	0.041 mA/mM	0.2–20 mM	3	I –V technique/Enzymatic	This work

*DR, Detection Range; DL, Detection Limit; RT, Response Time*.

## Conclusions

A novel applied GOx/nano-ZnO/PVA/FTO was reported to have high sensitivity and to be a powerful enzymatic biosensor in the measurement of glucose in human blood serum. The nano-material used in the design of GOx/nano-ZnO/PVA/FTO created a greater surface area for loading of enzyme that may improve the activity of the fabricated biosensor for determination of glucose. Because of strong enzyme–substrate covalent bonding we concluded that our fabricated biosensor had faster detection kinetics and reduced diffusion resistance in the measurement of glucose. The results obtained at the surface of the GOx/nano-ZnO/PVA/FTO showed a fast response time (less than 3 s) and a limit of detection of 2.0 μM with a sensitivity of 0.041 mA per mM for determination of glucose under optimum conditions. The GOx/nano-ZnO/PVA/FTO showed good selectivity with high sensitivity for determination of glucose in real-life samples such as human blood serum. The manufacturing process of the proposed biosensor is the future project of our research team.

## Data Availability Statement

The raw data supporting the conclusions of this article will be made available by the authors, without undue reservation.

## Ethics Statement

No potentially identifiable human images or data is presented in this study.

## Author Contributions

All authors listed have made a substantial, direct and intellectual contribution to the work, and approved it for publication.

## Conflict of Interest

The authors declare that the research was conducted in the absence of any commercial or financial relationships that could be construed as a potential conflict of interest.
